# InertialNet: Inertial Measurement Learning for Simultaneous Localization and Mapping †

**DOI:** 10.3390/s23249812

**Published:** 2023-12-14

**Authors:** Huei-Yung Lin, Tse-An Liu, Wei-Yang Lin

**Affiliations:** 1 Department of Computer Science and Information Engineering, National Taipei University of Technology, Taipei 106, Taiwan; 2 Department of Electrical Engineering, National Chung Cheng University, Chiayi 621, Taiwan; 3 Department of Computer Science and Information Engineering, National Chung Cheng University, Chiayi 621, Taiwan

**Keywords:** inertial measurement, visual inertial odometry, optical flow

## Abstract

SLAM (simultaneous localization and mapping) plays a crucial role in autonomous robot navigation. A challenging aspect of visual SLAM systems is determining the 3D camera orientation of the motion trajectory. In this paper, we introduce an end-to-end network structure, InertialNet, which establishes the correlation between the image sequence and the IMU signals. Our network model is built upon inertial measurement learning and is employed to predict the camera’s general motion pose. By incorporating an optical flow substructure, InertialNet is independent of the appearance of training sets and can be adapted to new environments. It maintains stable predictions even in the presence of image blur, changes in illumination, and low-texture scenes. In our experiments, we evaluated InertialNet on the public EuRoC dataset and our dataset, demonstrating its feasibility with faster training convergence and fewer model parameters for inertial measurement prediction.

## 1. Introduction

The objective of simultaneous localization and mapping is to understand how mobile robots can determine their own positions in an unknown environment and simultaneously build a consistent map [1]. It has been investigated over the past few decades, and is still considered as an unsolved and challenging problem [2]. Recently, due to the availability of low-cost sensing devices and the advances of visual-information-processing algorithms, computer vision and machine learning approaches have been used for the development of self-localization and 3D model reconstruction techniques [3]. A number of SLAM algorithms are applied to commercial applications, which means that mobile robots have the ability of autonomous navigation in structured environments such as offices and factories [4,5]. It is also a current research trend to adopt 3D optical sensors based on dynamic triangulation for the SLAM of a robotic swarm [6]. To deal with a group of robots for navigation, the idea of data transferring was proposed [7]. It is specifically important when the application environment contains a number of obstacles. Communication among the robots is crucial to the team during in situ operations.

The existing SLAM techniques which utilize the image data mainly consist of two approaches, namely visual simultaneous localization and mapping (visual SLAM) and visual odometry (VO). Under the general SLAM framework, visual SLAM uses the rich image information acquired from the environment [8]. On the other hand, visual odometry computes the motion trajectories of mobile platforms by analyzing the visual content derived using onboard cameras [9]. Compared to visual SLAM algorithms, which emphasize the globally consistent estimates and loop closures of the map, VO techniques adopt incremental pose updates based on the local consistency [10]. The main idea is similar to dead reckoning based on wheel odometry, but the image data obtained from exteroceptive sensors are used to mitigate the drifting problem [11].

Although vision-based approaches have been extensively studied, there are still very limited application scenarios incorporating visual SLAM due to the robustness issue [12]. The conventional feature-based methods are relatively stable under illumination changes, but the localization systems easily fail under low-texture surroundings [13,14]. In contrast, the direct methods are able to deal with low-texture scenes, but do not perform well if the invariant illumination is not satisfied (such as if the brightness of the scene is not fairly even due to the lighting condition or the surface reflectance property) [15,16]. One feasible solution to these robustness problems is through the development of visual–inertial odometry (VIO) techniques. In a VIO system, the information from the image sensor and IMU (inertial measurement unit) are integrated to obtain a better camera motion estimation [17]. This is based on the complementary characteristics of cameras and IMUs. The inertial measurement is able to provide the supplementary information when the visual tracking fails, but its high data rate usually suffers from the drifting problem. On the other hand, the drafting error can be greatly reduced by fusing the exteroceptive image data for the trajectory computation [18].

In recent progress, several VIO techniques have performed robust estimations from multi-sensor data fusion [19,20]. However, the robustness issues of monocular vision methods still require further investigation. The hand-crafted features commonly adopted in the computer-vision-based approaches do not provide sufficient information for the registration alignment. There will be great improvement in the stability of the visual SLAM systems if the features encoded in the images can be extracted comprehensively. In this paper, we employ a learning approach to model the relationship between the IMU sensor data and camera pose via image sequence analysis. An end-to-end deep neural network is proposed to predict the camera rotation while reducing the pipeline complexity of the architecture.

The existing end-to-end VO networks generally suffer from the generalization problem [21]. When the testing data appear very differently from the training images, the prediction accuracy degrades significantly. In this work, we present an end-to-end network structure to correlate the relationship between the image sequence and the IMU signals. The network model is constructed based on inertial measurement learning, and used to predict the pose of the general camera motion. Due to the model being designed with an optical flow substructure, the network is independent of the appearance of training sets and can be generalized to adapt to new environments. It is able to maintain a stable prediction under image blur, illumination changes and low-texture scenes. In the experiments, the proposed network is tested on the public EuRoC dataset and our dataset. The results demonstrate the feasibility of our InertialNet with faster training convergence and fewer model parameters for inertial measurement prediction. When the image sequences are captured from a new testing scene, our network model is able to predict the camera pose correctly.

The main contributions of this work are as follows.

- A new neural network structure, InertialNet, is proposed. It is designed for camera rotation prediction from image sequences, and the architecture is able to converge well and fast.
- The model generalization for new environment scenes is achieved via the architecture design with an optical flow substructure.
- Our proposed system is able to provide stable predictions under image blur, illumination change and low-texture environments.
- The EuRoC MAV dataset [22] is used for our evaluation. It is more challenging than the commonly adopted KITTI dataset used in previous works [23].

The rest of this paper is organized as follows. In Section 2, some related works from the literature are discussed. Section 3 presents our approach of an end-to-end model for IMU data prediction. The experimental results, as well as a performance evaluation, are provided in Section 4. Finally, in Section 5, some conclusions are drawn.

## 2. Related Work

The proposed InertialNet in this work is inspired by several machine-learning-based visual odometry methods. To estimate the camera pose from the acquired image sequences, the pixel relation between the consecutive frames should be established. The visual cues and geometric image formation are then used to compute the camera’s orientation. In the existing literature, visual odometry techniques based on deep neural networks can be divided into two categories. One approach directly uses the acquired image sequences for camera pose prediction [24,25], and the other utilizes the optical flow computation results to perform inference [26,27].

For the methods directly using the captured images as input, two sequential frames are stacked as a tensor. It is then taken into a convolutional neural network structure (such as AlexNet [28], VGG16 [29], GoogLeNet [30]). By modifying the hyper-parameters of the network layers close to the output, one can derive a six-vector representing the six-DoF camera pose. An important issue of this network design is the model generalization problem. The precision of the inference will be much lower if the network model is applied to a scene different from those in the training data. This is mainly because the network structures have learned both the pixel correspondences among the image frames and the global appearance of the scenes. To cope with this problem, a recurrent neural network (RNN) architecture is developed as a fusion framework for the IMU and laser data [31]. The robustness and accuracy of the robot pose estimation are then enhanced through scan-to-submap optimization. In [32], a 3D recovery system based on a multi-state constraint Kalman filter (MSCKF) is proposed. It utilizes the fusion of the visual and IMU data for an accurate localization of large-scale and long-range navigation.

The objective of the optical-flow-based approaches (such as VINet [27] and DeepVO [26]) aims to improve the generalization capability of the networks. In the model construction, the tensor generated from a stacked image sequence is used to compute the dense optical flow by FlowNet [33]. The tensor dimension is then reduced by CNN or RNN (recurrent neural network), and output to a six-vector via the fully connected (FC) layers. To serve as a substructure of the proposed InertialNet model, FlowNet and FlowNet2 [34] are currently the best ways to obtain the dense optical flow images. A structure based on the CNN model is used to train FlowNet with its own dataset. Compared to the previous models for optical flow estimation, FlowNet can provide lower loss and smoother predictions in a different scene. As a sequel to FlowNet, FlowNet2 consists of three sub-networks, FlowNetS, FlowNetC and FlowNetSD. The FlowNetSD structure is designed specifically for small displacement computation. By stacking all three sub-networks, much smoother and more precise predictions can be obtained.

In a more recent work, Liu et al. presented an unsupervised approach for optical flow estimation [35]. It utilized unlabeled image sequences with self-supervision from augmenting novel views. A forward pass was added to the basic learning network for transformed images to increase the reliability of view synthesis data. Xu et al. reformulated the optical flow prediction as a global matching problem, and proposed a transformer-based approach [36]. They used a refinement step to achieve better residual flow prediction. However, the technique does not generate well for the occluded regions and might introduce unreliable results. Similarly, a global matching method with patch-based overlapping attention was presented by Zhao et al. [37]. To improve the direct-regression optical flow estimation, an effective global matching step was introduced before optimization. In [38], consistency learning strategies were proposed for optical flow estimation. By utilizing the consistencies on occlusion and transformation, the technique is able to learn the description of pixel-level motion without additional annotation. Since the strategies can be applied to baseline models, the method can be used to improve the performance based on existing networks.

After years of handcrafted features used for visual odometry research, the end-to-end deep learning model provides a new paradigm for the pipeline design. However, despite the recent success of CNN-based classification techniques, most network structures are still not able to deal with a testing environment that is different from the training scenes. This situation usually occurs when a new application scenario is considered. To overcome this limitation, DeepVO is constructed in a way that the image appearance and the pixel motion can be learned simultaneously [26]. A CNN is adopted to extract the optical flow features and reduce the image dimension. The camera pose is then derived by modeling the sequential relation of the outputs using RNN and LSTM (long short-term memory). Although DeepVO can achieve good localization accuracy, the performance will drop significantly if there are dynamic objects contained in the scenes. Depending on the size of the dynamic region appearing in the image, the estimation error could be affected by a degree roughly proportional to the number of pixels. It is also very difficult to collect the ground truth data in broad areas. In [39], Zhu et al. presented an end-to-end network, DeepAVO, for visual odometry estimation. It consists of four parallel CNNs to learn four quadrants of optical flow with an attention module. Through the propagation of refined features and the concatenation of local cues on four branches, pose estimation was further improved.

VINet is the first deep-learning-based visual–inertial odometry technique [27]. Similar to DeepVO, the network model uses LSTM to process the input, but also includes the IMU data with much higher frequencies than the image frame rate. VINet concatenates the tensors from the images and IMU after synchronizing the signals. Consequently, the camera pose is predicted using LSTM with the output produced by the SE(3) concatenation layer. In a recent work, an ABRN (attention-based recurrent network) [25] incorporated an attention phase during graph-based optimization to develop a learning-based SLAM technique. The network is fully differentiable, and the accumulated errors of visual odometry predictions can be effectively reduced by the proposed neural graph optimizer. Nevertheless, ABRN is only tested for two-dimensional translation and yaw rotation with synthetic data only. More recently, Lu et al. integrated the camera pose graph optimization and bundle adjustment for network training in an unsupervised fashion [40]. In the proposed method, the pose drift was mitigated by motion and depth updates through pose graph and bundle adjustment optimization. This meant being able to train the network effectively via a selection of keypoints to optimize the camera poses.

Most deep-learning-based visual odometry techniques take the camera trajectory or depth images for supervised learning. The cost of data collection in a large-scale environment is extremely high. In this regard, UndeepVO adopts an unsupervised learning method and takes the unlabeled data for model training [41]. However, it is only tested on the KITTI dataset, and no experiments demonstrated this capability when applied to more complex camera motions. Li et al. presented an online adaptation framework using geometric information and Bayesian inference for deep visual odometry [42]. They adopted an online learned photometric uncertainty to optimize the depth and pose and to generalize the network for adaption to real-world scenes. In this study, we emphasize model generalization using the optical flow backbone. The network structure is constructed and the hyperparameters of the model are iteratively tuned through validation on the training data.

## 3. Approach

There are four primary objectives in the end-to-end model design of our proposed InertialNet: (1) the prediction speed, (2) the number of parameters, (3) the curve fitting ability, and (4) the model generalization. For the evaluation of our IMU signal prediction, we first define the three-axis rotation, yaw, pitch and roll by $\omega_{x}\left(t\right),\omega_{y}\left(t\right)$ and $\omega_{z}\left(t\right)$, and the RMS (root-mean-square) error at the time instant *t* is calculated as follows:

(1) $$RMS\left(t\right)=\sqrt{{\left(\omega_{x}^{p}\left(t\right)-\omega_{x}^{g}\left(t\right)\right)}^{2}+{\left(\omega_{y}^{p}\left(t\right)-\omega_{y}^{g}\left(t\right)\right)}^{2}+{\left(\omega_{z}^{p}\left(t\right)-\omega_{z}^{g}\left(t\right)\right)}^{2}}$$

where the superscripts *p* and *g* represent the prediction and groundtruth, respectively. Our proposed technique attempts to correlate the IMU data with the image information.

**Figure 1:**
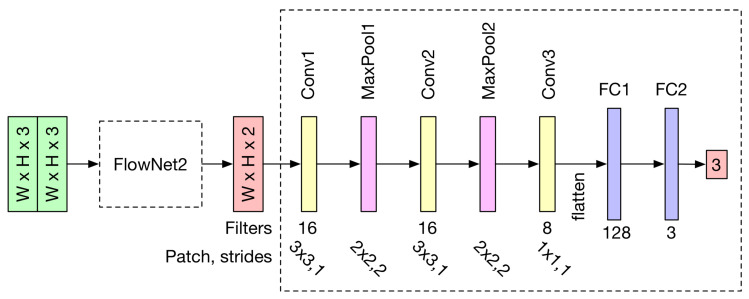
The proposed InertialNet architecture. The network model first takes the stacked images as input. The FlowNet2 substructure then converts the stacked images to optical flow images. The subsequent structure is responsible for transforming the optical flow information into IMU rotation data.

In general, a network model is more difficult to converge if there are too many layers or parameters. Thus, our network structure only adopts three convolutional layers and two max-pooling layers (with a stride of 2). To reduce the tensor size before the flattening, a 1×1 kernel is used for the convolutional layer Conv3 in Figure 1. Two fully connected layers (FC1, FC2) are then concatenated to reduce the tensor to a three-vector tensor for rotation prediction. To prevent the model from over-fitting and maintain the generalization ability, a 30% dropout is applied to the connection between Conv3 and FC1. This means that, at the training stage for each epoch, 30% of the neuron input will be randomly set to 0. Finally, to increase the penalty for large losses, the mean squared error is used as the training loss function.

To enhance computational speed, we take into account a more compact optical flow substructure. FlowNet is currently one of the best methods for obtaining a dense optical flow. It utilizes a CNN-like structure trained on its proposed dataset, which enables FlowNet to achieve a lower loss and smoother results in different scenes compared to other methods. To compare and evaluate different FlowNet approaches and implementations, we first examine the performance. Among the three sub-network variants, FlowNetC struggles to converge effectively on the EuRoC dataset due to its higher frame rate compared to the KITTI dataset. FlowNetSD, on the other hand, manages to converge on the EuRoC dataset but faces challenges with large disparities. To address these issues, we use FlowNet2 as the backbone of our network model. This helps us generate more stable optical flow images with reduced noise and improved convergence capabilities, particularly for handling the various disparities caused by camera motion.

After incorporating the optical flow substructure, the network predicts the camera pose based on the motion-encoded output images. Unlike previous visual odometry research, we do not directly use classic structures designed for general object classification tasks, as they have encountered convergence issues. Instead, we seek a model with sufficient fitting capabilities to predict all poses in the training set by exploring hyperparameters from simpler structures. To enhance the fitting capabilities of deep neural networks, one common approach is to add more convolutional and fully connected layers. However, this comes at the cost of slowing down network prediction due to the increased number of model parameters. In addition, the generalization ability of the model may suffer if the training data are not sufficient. In our proposed method, we adopt the following design principles to construct a suitable network structure.

During the process of deriving hyperparameters for InertialNet, it was noticed that the optical flow substructure could potentially lead to a same-value problem. Initially, the FlowNetC from DeepVO was utilized as the network backbone. However, the optical flow prediction from FlowNetC proved to be unstable when subjected to severe noise in the EuRoC dataset. The presence of noisy signals significantly affected the convergence of the network. Even when combining AlexNet with the FlowNet2 structure, the same-value output issue persisted, indicating that the problem might be attributed to the noisy data used for training the optical flow substructure. To address the noisy data problem during the optical flow substructure training, we developed a hyperparameter search method that involves adjusting AlexNet.

**Figure 2:**
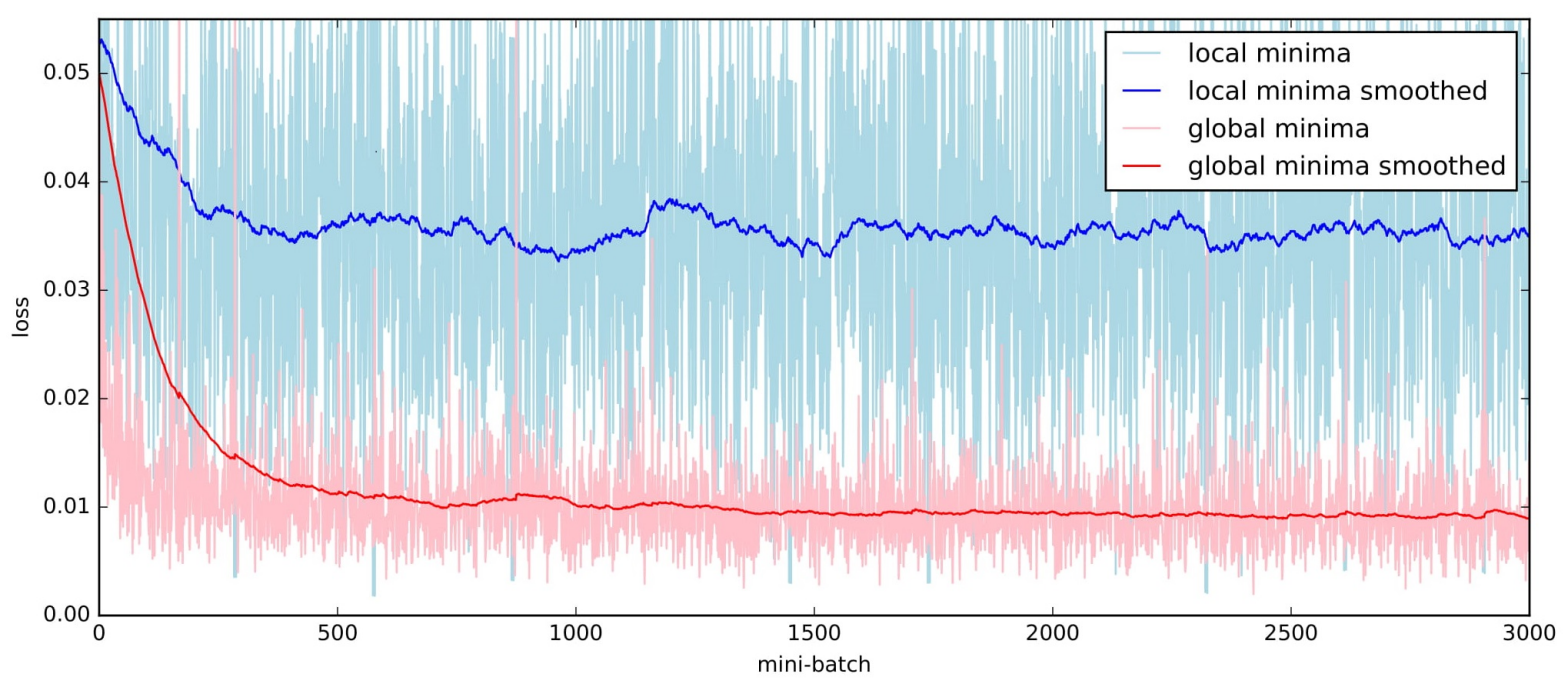
The training loss of the regression model for the V1_01_easy sequence in the EuRoC dataset. The original data points are plotted in light color, and the values after smoothing (smoothed=last×weight+(1−weight)∗current, weight=0.99) are indicated by the dark blue and red curves.

When we began searching for hyperparameters starting from a simpler structure, we observed that the Mean Squared Error (MSE) loss during the training phase converged faster (as shown by the red curve in Figure 2) and fell below the blue baseline. This indicated that the model was capable of predicting the camera rotation rather than just providing an average output value. Upon validation using the testing dataset, this model exhibited no same-value problem, and its prediction outputs were reliable. Compared to the current methods employing a Neural Architecture Search (NAS) [43,44], our proposed approach proved to be efficient and tailored for the specific three-vector prediction task. Furthermore, available NAS algorithms are predominantly designed for classification problems, leading to higher computational complexities. By building upon the optical flow backbone, our InertialNet architecture was ultimately constructed through parameter tuning and validation on the training set.

When using the convolutional layers to reduce the dimension of the input tensor, the size of the output tensor is given by

(2) $$\begin{array}{l} (N_{W},N_{H},N_{C})=\\ \;\;(\left\lfloor \frac{n_{W}+2p-f}{s}+1\right\rfloor ,\left\lfloor \frac{n_{H}+2p-f}{s}+1\right\rfloor ,n_{F}) \end{array}$$

where $n_{W}$ and $n_{H}$ are the width and height of the image input, respectively. $n_{F}$ is the number of convolution kernels, *p* is the padding pixel width and *f* and *s* are the size and the stride of the CNN kernels, respectively. In general, the adjustment of the stride has a significant impact on the dimension reduction. However, the image details will be missing if a too large a stride is used for the network. In our InertialNet, the convolutional layers with 1×1 kernels are adopted. This design is introduced in GoogLeNet, and does not reduce the dimensions of $N_{W}$ and $N_{H}$ [30]. The number of 1×1 kernels can be used to control the size of $N_{C}$ and increase the non-linearity of the network. As can be seen in the network structure illustrated in Figure 1, several max pooling layers are also applied to our InertialNet.

Compared to classification networks, observing the convergence of regression networks is more challenging. In classification problems, when the training loss reaches a specific value, it typically indicates that the network model can provide accurate predictions. However, in regression models, even if the training loss decreases to a certain level, it is still possible to have poor predictions. In such cases, the prediction output might be around the average and remain the same value for every input. To address this same-value problem in our network structure, we have incorporated the following strategy to prevent this issue from occurring.

Network training using two different sets of hyper-parameters is illustrated in Figure 2. First, the dataset EuRoC V1_01_easy is used to train our network model with arbitrary hyper-parameters, and we observe whether the loss is dropped when the number of layers or kernels are increased. If the same-values occur while tuning the hyper-parameters, the training loss (the blue curve shown in Figure 2) is taken as the baseline for our model training. The figure shows that, although the training loss (in MSE) indicated by the blue curve has reached 0.04, the network does not converge to the global minimum (as shown by the red curve).

## 4. Experiments

**Figure 3:**
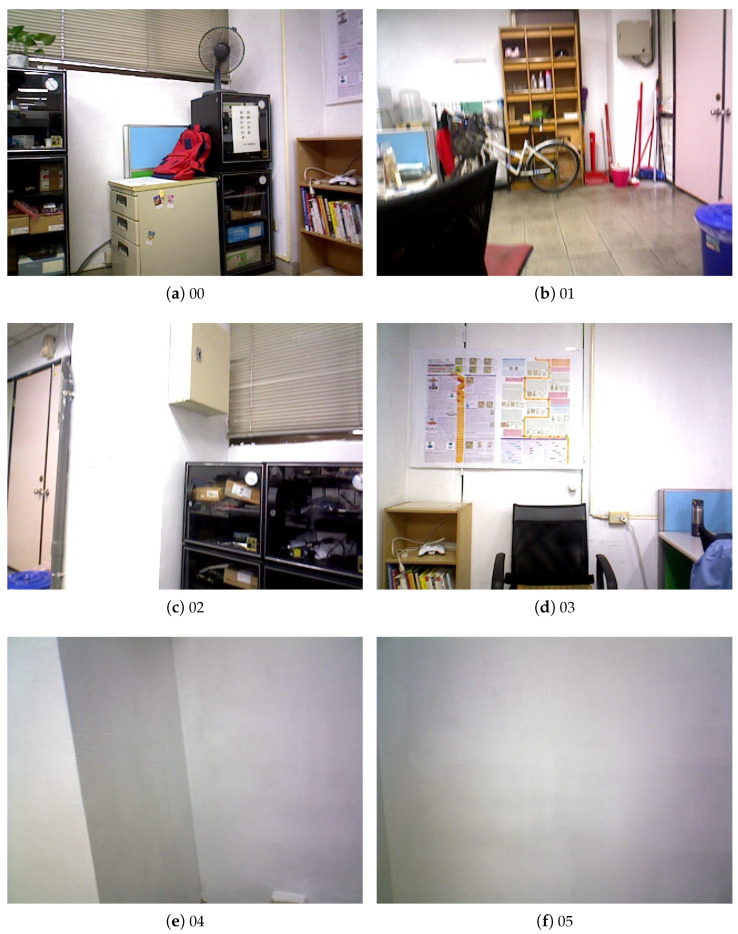
Some sample images in the InertialNet dataset. (**a**,**b**) translation and all rotation; (**c**,**d**) translation and horizontal rotation; and (**e**,**f**) white wall.

In our experiments, we use two datasets to test the IMU signals which are collected and serve as ground truth for evaluation. One is the public EuRoC MAV dataset [22], and the other is the InertialNet dataset collected by us in this work. For the EuRoC dataset, the image data are captured using a stereo camera system (MT9V034, WVGA, global shutter, 20 Hz) and the inertial data are collected by an IMU (ADIS16488, 200 Hz) mounted on a MAV (micro air vehicle). Our newly created InertialNet dataset consists of the images acquired using an ASUS Xtion Pro live RGB-D camera (30 fps) and synchronized with a low-noise IMU device (LORD MicroStrain AHRS 3DM-GX5-25). Some sample images in the InertialNet dataset are shown in Figure 3. These consist of several indoor scenes captured with the camera translation and rotation motion.

### 4.1. IMU and Image Synchronization

**Figure 4:**
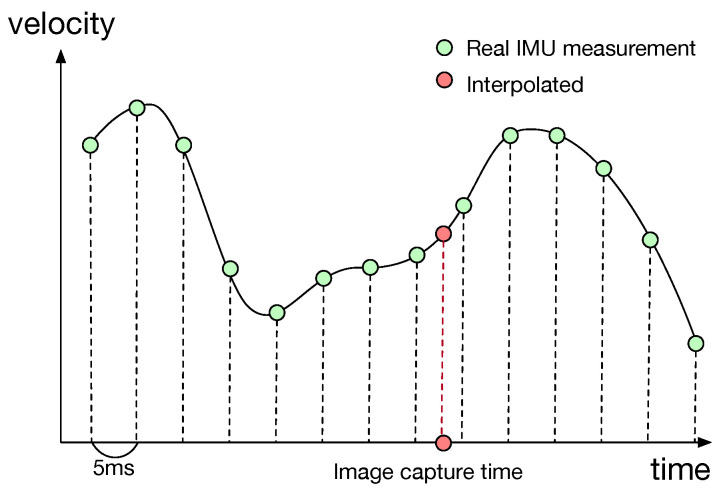
The IMU data and image sequences in the EuRoC dataset are not synchronized. We take the IMU data points to create a cubic spline model to interpolate the IMU signals for alignment with the camera recording.

Since the sampling rates are different for the recording of image and IMU data, the signals from multi-modality sources are required to be synchronized prior to further processing. For the EuRoC dataset, we take the IMU data points to create a cubic spline model. It is then used to perform interpolation for signal alignments based on the image acquisition time, as illustrated in Figure 4. We only use the synchronized IMU and image data for network training in the experiments.

The implementation of our InertialNet network architecture is carried out using PyTorch 0.4.0. It is carried out on the hardware with an NVidia GeForce GTX 1080 graphics card and an Intel Core i7-6700K 4.0 GHz CPU with 32 GB of main memory. We use the Ubuntu16.04 operating system for the implementation. The memory requirements for FlowNet2, FlowNetSD and InertialNet (without the FlowNet’s weights) are 650 MB, 181 MB and 92 MB, respectively. The stochastic gradient descent (SGD) is adopted to minimize the loss function. We set the learning rate as $10^{-6}$, and the network model is trained using a mini-batch size of 10 with 20–30 epochs.

The EuRoC dataset records grayscale images and the resolution is 752×480×1 (single channel). Under FlowNet2 data encoding, the image resolution becomes 752×480×2. In our InertialNet dataset, the color images are acquired with a resolution of 640×480. It has the output image size of 640×480×2 after processing by FlowNet2. The statistics of the EuRoC and InertialNet datasets are tabulated in Table 1 and Table 2, respectively. These contain the sequences of different camera motion speeds in the EuRoC dataset. The dataset also consists of some sequences (MH_01–MH_05) with low illumination scenes. To increase the variety of data for a more comprehensive investigation, our InertialNet dataset aims to collect the data under a low-texture background and pure camera rotation motion. They are mainly used to test the model robustness and generalization capability.

### 4.2. Prediction Results

The robustness and precision of the InertialNet is evaluated using the following three metrics:

- The rotation error for time *t*.
- The prediction RMS error (root-mean-square error).
- The distribution of the prediction errors.

The EuRoC and our InertialNet datasets are used to train the network model separately since they have different IMUs and cameras for data acquisition. For the EuRoC dataset, we take the sequence V1_01 for model training, and use the remaining data for testing. As for our InertialNet dataset, the sequence 00 is used for training, and six additional sequences, 01 to 06, are used for testing.

**Figure 5:**
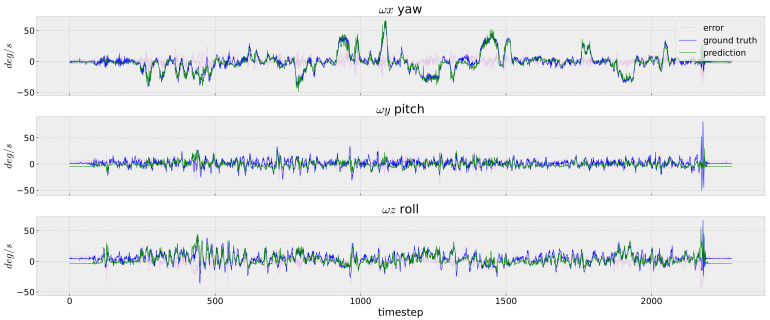
IMU rotation prediction. EuRoC V2_01_easy. The image sequence captured by the camera; simple motion in a room. InertialNet is able to correctly predict the rapid motion in the $w_{x}$ axis.

**Figure 6:**
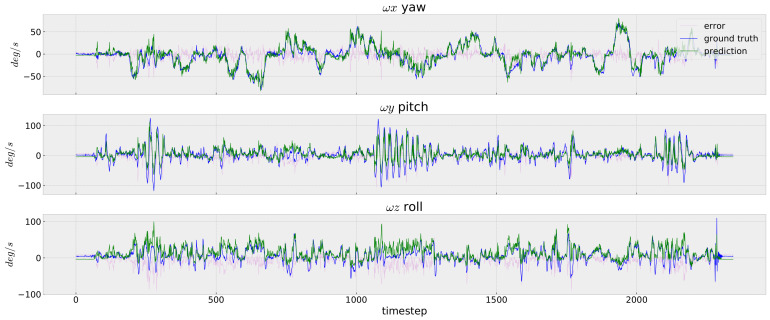
IMU rotation prediction. EuRoC V2_02_medium. The sequence is recorded in a different place from the training set. It can be seen that InertialNet is properly generalized to predict the correct rotation trend in a new environment.

**Figure 7:**
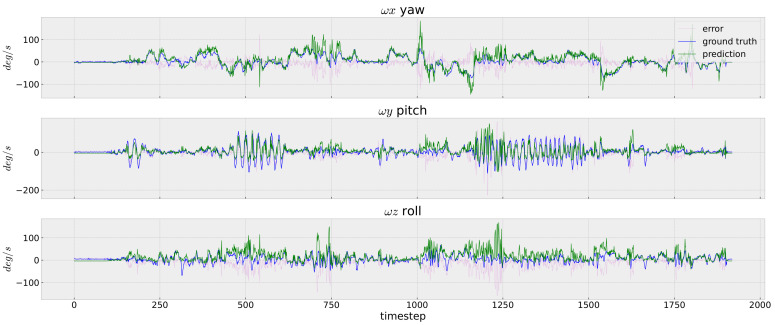
IMU rotation prediction. EuRoC V2_03_difficult. Although InertialNet is trained with the image sequences with simple motion, it is able to predict the trend of rapid motion. Nevertheless, there exist large errors at around t=1250.

In Figure 5, Figure 6 and Figure 7, we depict the prediction errors under each time-step of the testing data V2_02 for the easy, medium and difficult sequences, respectively. Although the network is trained using only the V1_01 sequence, our InertialNet can still provide the correct trends on the rotation prediction for V2_02 with faster camera motion. Nevertheless, the rotation angles on the pitch and roll directions are less accurate within the time-steps from 1100 to 1300. The apparent errors on the EuRoC testing data could be due to the lack of rapid-motion data collected in our InertialNet training set. For the different scenes in the factory environment, the proposed InertialNet is still able to obtain an accurate prediction, as the results of the EuRoC sequences MH_01–MH_05 show (Table 1).

**Figure 8:**
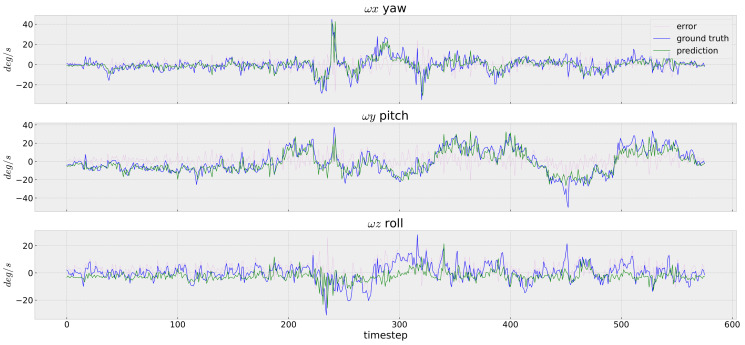
The IMU rotation prediction of the sequence ‘04 white wall’ in our dataset. The proposed InertialNet performs well in this low-texture scene. Compared to the EuRoC datasets, our captured data are less noisy and the curves appear smoother.

For the experiments carried out on our InertialNet dataset, two especially difficult examples are shown in Figure 3e,f. These are images from the ‘04’ and ‘05’ white wall sequences, which contain indoor scenes with much less texture. In the captured images, there are only some smooth intensity variations on the white walls. The results in Figure 8 illustrate that, even in this challenging case, the green prediction curves obtained from our InertialNet are very close to the blue ground-truth curves.

The prediction RMS errors of several image sequences in the EuRoC dataset, and our InertialNet dataset, are tabulated in Table 1 and Table 2, respectively. The evaluation results in Table 1 demonstrate that we are able to achieve RMS errors lower than 10 degrees, even when the training set V1_01 contains images with very different appearances from the test sequences V2_01 and MHs. It also shows that the V2_02 and V2_03 sequences have higher RMS errors. These results are due to the lack of rapid camera motion sequences in the training set V1_01. Thus, our InertialNet provides a model generalization capability above a certain level. Furthermore, as the results show in Table 2, the RMS errors of the white wall sequences in the InertialNet dataset are under 7 degrees. We may conclude that our model is able to provide the same prediction accuracy under generally less textured environments.

**Figure 9:**
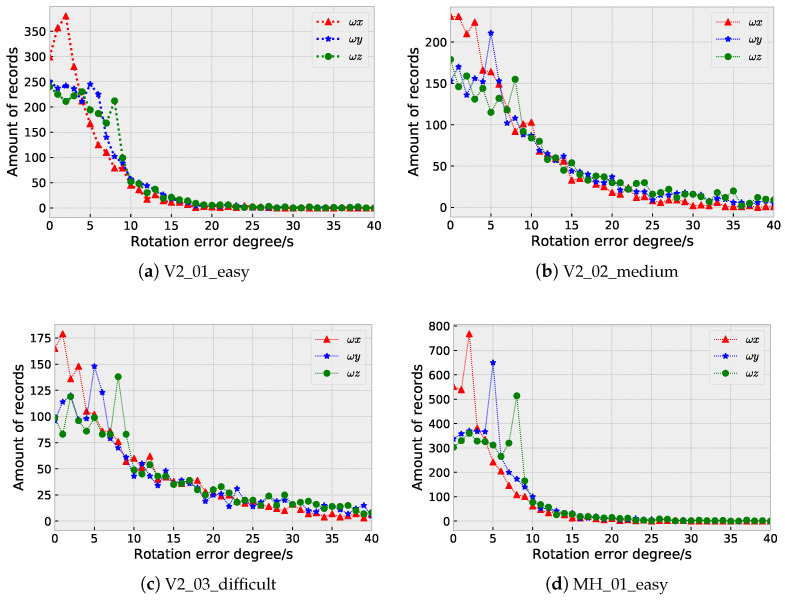
The distributions of the rotation prediction errors (in degree) using InertialNet for the sequences in the EuRoC dataset.

**Figure 10:**
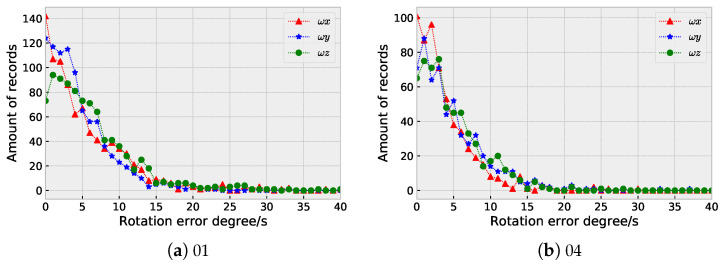
The distributions of the rotation prediction errors (in degree) using InertialNet for the sequences in our dataset.

Figure 9 and Figure 10 illustrate the distributions of the rotation prediction errors for several sequences in the EuRoC and our InertialNet datasets, respectively. The results indicate that most of the errors are less than 15 degrees. To deal with the large errors that appeared in V2_02_medium (Figure 9b) and V2_03_difficult (Figure 9c), it is necessary to include more data acquired with different camera poses for network training.

### 4.3. Comparison with Similar Approaches

**Figure 11:**
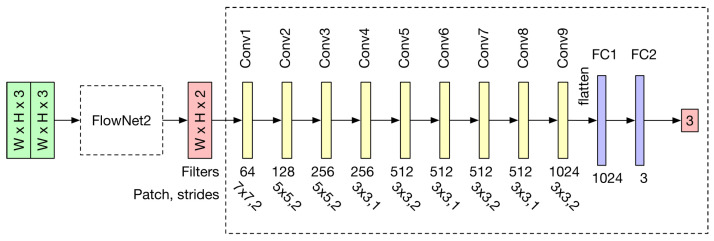
The Flowdometry [45] structure with a minor modification. Although the architecture is similar to our InertialNet, the Flowdometry model is not able to converge on the EuRoC and InertialNet datasets.

**Figure 12:**
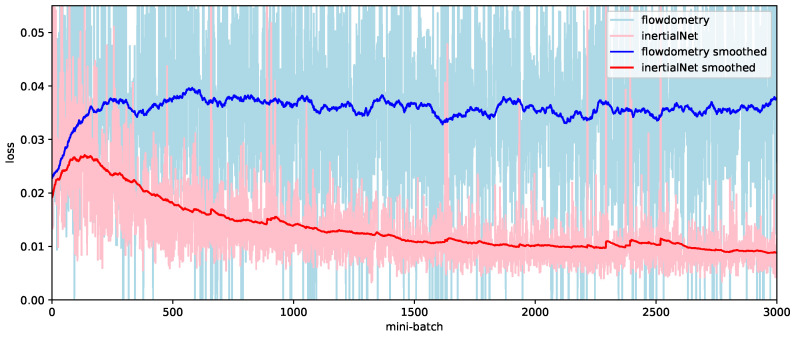
The training loss comparison of the Flowdometry and InertialNet models. The training loss of Flowdometry does not decrease for more than 3000 epochs.

To the best of our knowledge, there are no other works in the existing literature which have addressed the IMU data prediction problem directly from the image sequences. Thus, we made some changes to our original architecture to compare it with related studies. In a previous work [45], Flowdometry demonstrated a network structure relatively close to ours. The stacked images are used to predict the six-DoF camera pose of the motion trajectory. As illustrated in Figure 11, FlowNet2 is adopted as the backbone, and the FC2 layer is slightly adjusted from six nodes to three. Although the network structures are similar, Flowdometry is not able to converge on the EuRoC dataset as well as the InertialNet model. Figure 12 illustrates the training loss comparison of Flowdometry and InertialNet. The blue curve of Flowdometry has much larger variation than the red curve of InertialNet. Furthermore, the training loss generally remains at a high level, and does not decrease even when Flowdometry is trained with more epochs on the EuRoC dataset.

## 5. Conclusions

To enhance the robustness of SLAM systems, we present an end-to-end network model to derive the relationship between the image sequence and IMU signals. The proposed InertialNet model is able to provide stable camera pose prediction even under different motion trajectories and low-texture scenes. It is the first attempt to learn the IMU data from image sequences using a deep neural network. Due to its architecture design with an optical flow substructure, the model is generalized to adapt to new environments successfully. In the experiments carried out on real-world scenes, the proposed network model was tested on the public EuRoC dataset and our InertialNet dataset. The results demonstrated the feasibility of our method for inertial measurement learning and its capability in model generalization.

## Figures and Tables

**Table 1:** The IMU data and image sequences of the EuRoC dataset used in our experiments. The numbers of IMU (before the synchronization) and Cam0 recordings are listed. We only use the sequence V1_01 to train our InertialNet model. The rest of the sequences are used for testing. The names starting with V and MH are recorded in the room and factory environments, respectively.

Seq. Name	# of Images	IMU	RMSE (deg) (wx, wy, wz)
V1_01	2912	29,120	for training
V1_02	1710	17,100	10.16, 12.92, 15.41
V1_03	2149	21,500	10.85, 20.12, 18.95
V2_01	2280	22,800	5.97, 7.52, 7.64
V2_02	2348	23,490	10.50, 17.88, 17.32
V2_03	1922	23,370	18.52, 26.81, 25.10
MH_01	3682	36,820	5.93, 8.75, 8.50
MH_02	3040	30,400	7.09, 8.91, 9.36
MH_03	2700	27,008	7.61, 9.79, 9.21
MH_04	2033	20,320	6.51, 7.92, 7.91
MH_05	2273	22,721	6.06, 7.72, 6.71
Total	27,049	274,649	

**Table 2:** There are seven IMU data and image sequences recorded in our InertialNet dataset. The numbers of images and IMU data recordings are the same since we have synchronized the sensors for data collection.

Seq.	Records	Content	RMSE (deg) (wx, wy, wz)
00	1140	all rotation and translation	for training
01	905	all rotation and translation	9.11, 6.46, 9.14
02	1126	pure rotation	3.95, 6.09, 4.48
03	1183	pure rotation	5.01, 8.72, 4.94
04	578	white wall	5.64, 7.01, 6.59
05	1094	white wall	5.25, 7.24, 6.47
06	858	all rotation and translation	8.45, 9.35, 6.75
Total	6884		

## Data Availability

Data are contained within the article.
